# Intensive pulmonary rehabilitation in a pediatric lung transplantation patient: A case report

**DOI:** 10.1097/MD.0000000000025523

**Published:** 2021-04-30

**Authors:** Eun Jung Choi, Won Kim, Jae Yong Jeon, Eun Jae Ko, Jinho Yu, Se Hoon Choi, Seung Hak Lee, In Young Sung

**Affiliations:** a Department of Rehabilitation Medicine, Asan Medical Center, University of Ulsan College of Medicine, Seoul; b Department of Rehabilitation Medicine, Seongnam Citizens Medical Center, Seongnam; c Department of Pediatrics, Asan Medical Center, University of Ulsan College of Medicine, Seoul; d Department of Thoracic and Cardiovascular Surgery, Asan Medical Center, University of Ulsan College of Medicine, Seoul, Republic of Korea.

**Keywords:** exercise, lung transplantation, pediatric, rehabilitation

## Abstract

**Background:**

The pediatric lung transplant is a very important treatment for patients with end-stage lung diseae, and pulmonary rehabilitation (PR) is also an important factor in determining the prognosis. However, there is no much literature available on pulmonary rehabilitation in pediatric patients’ post lung transplant. Through this case report, we would like to present our intensive PR program for pediatric patients’ post-lung transplant.

**Patient concerns:**

The 10-year-old boy's breathing before receiving a lung transplant continued to deteriorate and he eventually became dependent on a wheelchair.

**Diagnosis:**

He was diagnosed with infantile acute lymphoblastic leukemia at 6 months of age. At the age of one year, he underwent allogeneic bone marrow transplantation, but was diagnosed with post-transplantation bronchiolitis obliterans (PTBO) two months later. He had a lung transplant at the age of 10.

**Interventions:**

He was hospitalized and received an initial assessment. This assessment included functional, cognitive, and psychological evaluations. He additionally completed PR exercises twice daily for two weeks. After discharge, he continued to participate in an outpatient-based PR program for three months. During the outpatient phase, PR exercises were performed once weekly, in addition to home-based cognitive training.

**Outcomes:**

Our intensive post-lung PR program improved our patient's exercise capacity, lung function, and quality of life. As a comprehensive rehabilitation service, our program also included a cognitive training component.

**Conclusion:**

We describe an intensive PR program tailored to pediatric patients’ post-lung transplant. The program was feasible and resulted in improvements in functional exercise capacity, lung function, and quality of life. Future research into our method is necessary for continued improvement of this novel program.

## 1 Introduction

Lung transplant improves quality of life and survival in patients with end-stage lung diseases.^[1]^ Unfortunately, many lung transplant recipients exhibit persistent impairments in exercise capacity and skeletal muscle function, despite having experienced significant improvements in lung function after surgery.^[2]^ Adults who undergo pulmonary transplants followed by exercise training showed improved motor function^[3]^ and a reduced incidence of transplant-related morbidities such as osteoporosis.^[4]^

In children, pediatric lung transplant is a viable option for the treatment of end-stage lung disease. According to the International Society for Heart and Lung Transplantation (ISHLT) registry, more than 2200 pediatric lung transplants have been reported since 1986. During the early 2000 s, the number of pediatric lung transplant patients increased steadily, with 125 reported to the ISHLT in 2009.^[5]^ Diagnoses that commonly lead to lung transplants in children include cystic fibrosis, pulmonary hypertension, congenital heart disease, and bronchiolitis; these diagnoses are different for adults, and they vary by age.^[6–8]^ Moreover, pediatric patients have additional issues related to development, growth, and education. Therefore, rehabilitation strategies for pediatric patients’ post-lung transplant should be comprehensive and modified to differ from programs used in adults. There are very few reports on pulmonary rehabilitation (PR) in pediatric patients’ post-lung transplant. The purpose of this case report was to describe our intensive PR program for pediatric patients’ post-lung transplant.

## 2 Case description

The patient was born in 40 weeks gestational age and weighed 4000 grams. His was born normal, and there were no abnormal findings at birth or during his prenatal examination. He was diagnosed with infantile acute lymphoblastic leukemia at six months of age and treated with allogeneic bone marrow transplantation at one year of age. Two months after the transplant, he was diagnosed with post-transplantation bronchiolitis obliterans (PTBO). His breathing worsened, and he became dependent on a wheelchair for transport. Spirometry at age seven—on April 11, 2016—showed a mixed pattern with a forced expiratory volume in 1 sec (FEV_1_) of 0.20 L; FEV_1_% predicted = 23%; forced vital capacity (FVC) = 0.23 L; FVC % predicted = 24%; and FEV_1_/FVC = 98%. Approximately six months later, he began using 24-h home ventilation, and his doctor recommended a lung transplant. He underwent chemotherapy due to recurrent leukemia and was a candidate for lung transplant in August 2018, at age nine. He underwent lung transplant surgery 10 months later. He had been taking steroids after being diagnosed with PTBO, and immune-suppressants were added after lung transplant. He underwent three weeks of 20-min sessions of bedside physical therapy, three times per week in the intensive care unit. These sessions included range-of-movement (ROM) exercises, breathing exercises, and bed-positioning education. He was later discharged and visited the Department of Rehabilitation Medicine while still using a wheelchair.

**Figure 1:**
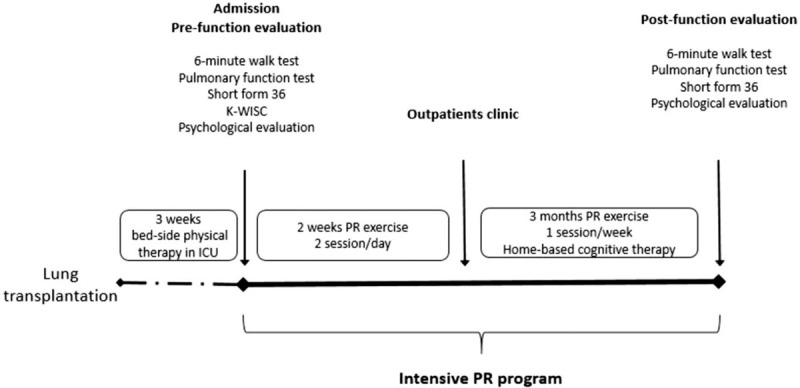
Flowchart of the intensive pulmonary rehabilitation program for pediatric patients post-lung transplant. ICU = intensive care unit, K-WISC = Korean Wechsler intelligence scale for children, PR = pulmonary rehabilitation.

Three months after surgery, he was hospitalized for two weeks and underwent various assessments before starting an intensive PR program (Fig. 1). These assessments included a comprehensive musculoskeletal assessment (muscle strength, posture, joint ROM), functional assessments (mobility skills of transfer, gait, and stair climbing), and functional exercise capacity assessments with the 6-minute walk test (6MWT), lung function test (FEV_1_, FVC), and quality of life evaluation [Short Form (SF)-36]. All of the above assessments were followed up during the outpatient phase.

At the time of admission, the patient's manual muscle test results were grade 4 in all extremities. His functional ambulatory category was stage 4, his pediatric balance scale score was 16 s, and he completed the sit-to-stand test in 16 s. During hospitalization, he was encouraged to participate in two weeks of PR exercises, twice daily for 1 h per session. These included breathing, aerobic, resistance, and flexibility exercises and gait training. Aerobic training involved a treadmill exercise with a speed of 0.7 km/h and a 10% incline for 20 min. The patient was allowed to exercise for 20 min until his modified Borg dyspnea scale (MBS) score was lower than three points. Cycle aerobic training (with a resistance of 2/20) was maintained for 20 min with a MBS score less than three points. After an intensive PR program of more than 3 months, he achieved a speed of 1.5 km/h and 10% incline for 20 min during aerobic training and a resistance of 4/20 during cycle aerobic training.

Cognitive function and psychologic assessments were also completed. The Korean Wechsler intelligence scale for children (K-WISC) showed a moderate cognitive impairment score of 59 points on the Full-Scale Intelligence Quotient. Depression and anxiety were elevated to subclinical ranges on the patient's psychological evaluation. He reported that he was less likely to enjoy himself and whether sad or depressed, scared or worried, and become nervous too often. After the psychological evaluation, we consulted with his parents about a possible psychological intervention; however, both the patient and his parents refused any psychological interventions.

Comprehensive rehabilitation goals were established with attention to the patient's motivation and the caregiver's goals. The patient's mother wanted him to go to school because he had never been to school. Our short-term goal was to increase the patient's ambulatory function. The patient's long-term goal was to attend school. After discharge, he participated in an outpatient-based PR program for three months. The program included once-weekly PR exercises, which were similar to the inpatient PR exercises; however, during outpatient PR, we gradually increased the exercise intensity according to the patient's improvement. We also initiated a home-based cognitive program using tablet personal computer during the outpatient phase. This cognitive program was used three times per week, for 30 min per day, for a total of three months.

The patient's adherence to intervention was good.

Before PR, the patient's 6MWT scores showed a reduction in functional exercise capacity, in comparison with predicted values (6MWT distance = 165 m; 35% predicted value).^[9]^ The patient reported improved dyspnea during the 6MWT and increased his distance walked by 75 m (6MWT distance post-rehabilitation = 240 m; 51% predicted; change (Δ) in meters walked = 45%). The patient's spirometry results also demonstrated improvements in FVC and FEV_1_ (FVC pre-PR and post-PR = 0.78 L and 1.04 L, respectively; Δ FVC + 33.3%). On the SF-36, the physical component summary showed a large improvement over the mental component summary (Table 1). Both the patient and his parents reported a high level of satisfaction with the home-based cognitive program. However, we did not perform a follow-up cognitive evaluation after the program. The patient planned to enter school in March 2020. However, this was postponed due to the novel coronavirus outbreak in the spring of 2020. There were no adverse and unanticipated events in this study.

**Table 1:** Functional parameters before and after the PR program.

	Pre-OP	Pre-PR	Post-PR
6MWT, m	–^∗^	165 m	240 m
FVC, L/min	0.22	0.78	1.04
FEV_1_, L/min	0.17	0.58	0.81
SF-36, Physical component summary (%)	–^∗^	51.25	77.5
SF-36, Mental component summary (%)	–^∗^	71.75	79.37

## 3 Discussion

Our pediatric case showed improvements in functional exercise capacity, lung function, and quality of life after participating in the intensive PR program following lung transplant. Several studies have described exercise-based PR programs for adults^[10,11]^; however, there is very little published literature that focuses on the pediatric population. Burton et al described that pediatric patients’ post-lung transplant who participated in a 1-h exercise training session, three times per week, at an outpatient clinic for three months, showed significant physical recovery.^[12]^ Our intensive PR exercise appeared to facilitate functional improvements in our patient. Exercise training once per week during the outpatient phase would not have been sufficient for this patient. However, the patient and his caregivers were provided with intensive exercise training for rehabilitation during his two-week hospitalization, and home-based exercises were well performed during the outpatient phase. Even though previous studies and our case demonstrated functional improvements after physiotherapy and PR, there is no consensus on the best techniques and regimens of PR exercises in pediatric patients’ post-lung transplant. Therefore, large-scale prospective clinical studies are necessary in the future.

We developed a specialized PR program for pediatric patients. This program included both physical therapy and psychocognitive components and reflected our patient's individual rehabilitation goal of attending school. Our patient, who had no brain lesions, exhibited a moderate cognitive impairment on the K-WISC. Piasecki et al found that children with cystic fibrosis and inflammatory bowel disease had lower attention and memory tests scores than healthy controls, with more profound cognitive impairments seen in the cystic fibrosis group.^[13]^ The patient was unable to attend “regular” school due to his chronic lung disease. Long-term steroid use may also produce adverse neuroanatomic and functional changes and may induce toxicity.^[14]^ Although the patient and caregivers were satisfied with the home cognitive program that used a tablet personal computer, further investigations are necessary to evaluate its efficacy. An estimated 4–11% of school-aged children develop depression within three months of being diagnosed with a medical illness.^[15]^ Chronic medical illnesses and their psychosocial correlations are associated with specific stages of development and may vary depending on socioemotional challenges.^[16]^ Although our patient did not receive psychotherapy, psychosocial support should be included in intensive PR programs for these patients.

Our rehabilitation program may be improved in the future. PR exercises before lung transplantation—so-called prehabilitation, are beneficial in adults.^[17]^ Our program should include PR exercises during the preoperative phase. Another issue involves differentiation of the PR program according to the patient's age. Rehabilitation demands and goals depend on the age of the pediatric patient, and age should be carefully considered when designing PR programs. In terms of growth and development in pediatric patients, long-term follow-up of patients who participate in PR programs is necessary.

## 4 Summary

In conclusion, an intensive PR program for pediatric patients’ post-lung transplant is feasible and necessary. Such a program should be comprehensive and include various aspects of rehabilitation, including cognition, psychology, and education. Further clinical investigations are necessary to determine the efficacy of this program and additional program modifications will be done if needed.

## Acknowledgments

I am extraordinarily thankful to my advisor, Junekyung Lee, and unwavering support and encouragement throughout this process.

## Author contributions

**Conceptualization:** Eun Jung Choi, Won Kim, Jae Yong Jeon, Eun Jae Ko, Jinho Yu, Se Hoon Choi, Seung Hak Lee, In Young Sung.

**Data curation:** Eun Jung Choi, Won Kim, Jinho Yu, Se Hoon Choi, Seung Hak Lee, In Young Sung.

**Formal analysis:** Eun Jung Choi, Seung Hak Lee.

**Investigation:** Eun Jung Choi, Won Kim, Jinho Yu, Seung Hak Lee.

**Methodology:** Eun Jung Choi.

**Project administration:** Eun Jung Choi, Jinho Yu, Se Hoon Choi, Seung Hak Lee, In Young Sung.

**Resources:** Seung Hak Lee, In Young Sung.

**Supervision:** Won Kim, Jae Yong Jeon, Eun Jae Ko, Jinho Yu, Se Hoon Choi, Seung Hak Lee, In Young Sung.

**Validation:** Eun Jung Choi.

**Visualization:** Eun Jung Choi.

**Writing – original draft:** Eun Jung Choi, Seung Hak Lee, In Young Sung.

**Writing – review & editing:** Eun Jung Choi, Seung Hak Lee, In Young Sung.
